# The association of two polymorphisms in adiponectin-encoding gene with hypertension risk and the changes of circulating adiponectin and blood pressure: A meta-analysis

**DOI:** 10.18632/oncotarget.14680

**Published:** 2017-01-16

**Authors:** Jianmin Wu, Guoyan Xu, Wenqin Cai, Yun Huang, Ningyu Xie, Yihua Shen, Liangdi Xie

**Affiliations:** ^1^ Department of Cadre's Ward, The First Affiliated Hospital of Fujian Medical University, Fuzhou, Fujian, China

**Keywords:** hypertension, adiponectin, polymorphism, blood pressure, meta-analysis

## Abstract

**Objectives:**

This meta-analysis was prepared to synthesize published data on the association of two polymorphisms (T45G and G276T) in adiponectin-encoding gene (*ADIPOQ*) with hypertension risk and the changes of circulating adiponectin and blood pressure.

**Methodology and Major Findings:**

Data were collected and corrected by two authors, and were managed with Stata software. In total, 12 articles were synthesized, including 12 studies (3358 cases and 5121 controls) for the association of two study polymorphisms with hypertension risk and 11 studies (3053 subjects) for the between-genotype changes of adiponectin and/or blood pressure. Based on all qualified studies, the risk prediction for hypertension was nonsignificant for both polymorphisms, with significant heterogeneity for G276T polymorphism (*I*^2^ = 53.8%). Overall changes in adiponectin and blood pressure were also nonsignificant for T45G, while contrastingly 276GT genotype was associated with significantly higher levels of adiponectin (weighted mean difference [WMD] = 0.72 μg/mL, 95% confidence interval [CI]: 0.04 to 1.41, *P* = 0.038), systolic (WMD = 5.15 mm Hg, 95% CI: 0.98 to 9.32, *P* = 0.016) and diastolic (WMD = 3.45 mm Hg, 95% CI: 0.37 to 6.53, *P* = 0.028) blood pressure with evident heterogeneity (*I*^2^ = 72.0%, 78.3% and 80.0%, respectively), and these associations were more obvious in hypertensive patients. Publication bias was a low probability event for overall comparisons.

**Conclusions:**

Our findings suggested that in spite of the nonsignificant association between *ADIPOQ* T45G or G276T polymorphism and hypertension, the heterozygous mutation of G276T was observed to account for increased levels of circulating adiponectin and blood pressure, especially in hypertensive patients.

## INTRODUCTION

Adiponectin, secreted exclusively by adipocytes, is a collagen-like protein containing 247 amino acids in length [1]. There is ample *in-vitro* evidence sustaining a physiologic role of adiponectin in the amelioration of endothelial function and the stimulation of nitric oxide production, as well as the regulation of body metabolism and immune responses [2–5]. Circulating adiponectin concentration is relatively high (5 to 30 μg/mL) and is inversely associated with blood pressure and the future risk of hypertension [6–8]. It is worth noting that human adiponectin concentration is predominantly under genetic control, with an estimated heritability of 40–70% [9]. The gene encoding adiponectin namely *ADIPOQ* (Gene ID: 9370) is mapped on chromosome 3q27 and is composed of 3 exons. The coding sequences of *ADIPOQ* contain 1273 validated single nucleotide polymorphisms (http://www.ncbi.nlm.nih.gov/gene/). In *ADIPOQ*, two polymorphisms, T45G (rs2241766) in the 2^nd^ exon and G276T (rs1501299) in the 2^nd^ intron, have been widely investigated in association with hypertension risk and circulating adiponectin changes [10, 11]. However, the results are not often repeatable. For example, in a Hong Kong population, the 45TT genotype was significantly correlated with a lower circulating adiponectin concentration [12], while no correlation was identified in a Finnish population [10]. In addition, carriers of the 276T allele had a 56% increased risk of hypertension in Jordanians [13], yet a 46% reduced risk in Taiwanese [14] when compared with the 276GG homozygotes. The conflicting findings may mirror the divergences in ethnicity, genetic profile, outcome definition or study power [8, 15]. As such, a comprehensive pooled analysis will be highly recommended to help interpret these divergences. Given the methodological heterogeneity and limited power of previous individual studies, we here carried out a meta-analysis to synthesize published data on the association of T45G and G276T polymorphisms in *ADIPOQ* with the risk of hypertension and the changes of circulating adiponectin and blood pressure. Meanwhile, we intended to track certain potential sources of heterogeneity between available studies.

## RESULTS

### Qualified studies

After rigorous literature search, a total of 41 articles were found, wherein 12 articles were qualified for the current meta-analysis [10–14, 16–22]. Out of 12 qualified articles, 8 articles including 12 independent studies (3358 cases and 5121 controls) compared genetic data on T45G and/or G276T polymorphisms between hypertensive and normotensive subjects [12–14, 16–19, 21], and 7 articles including 11 independent studies (3053 subjects) compared mean values of circulating adiponectin and/or blood pressure across the genotypes of polymorphisms under study [10–12, 14, 16, 20, 22].

### Study characteristics

Supplementary Table 1 shows the basic characteristics of 12 qualified studies for hypertension risk. Nine studies were carried out in Chinese, 2 studies in Jordanians and 1 study in Japanese. Eight studies had population-based controls and 4 studies had hospital-based controls. Two studies enrolled hypertensive patients complicated with metabolic syndrome, 2 studies with type 2 diabetes mellitus, 1 study with coronary artery disease and 1 study with obesity. Age was reported to be comparable between hypertensive and normotensive subjects in 7 studies. The genotypes of study polymorphism(s) were determined by TaqMan technique in 5 studies, by restriction fragment length polymorphism (RFLP) technique in 4 studies, by MassARRAY technique in 2 studies and by direct sequencing in 1 study. Total sample size of 12 qualified studies ranged from 213 to 1616.

Of 11 qualified studies for the changes of circulating adiponectin and/or blood pressure, 5 studies exhibited these changes in hypertensive patients, 3 studies in normotensive controls, 2 studies in combined subjects and 1 study in patients with primary aldosteronism. Circulating adiponectin concentration was measured mainly by enzyme linked immunosorbent assay (ELISA) kit (*n* = 9 studies), and 2 studies adopted sandwich enzyme immunoassays.

### Overall analyses

**Figure 1:**
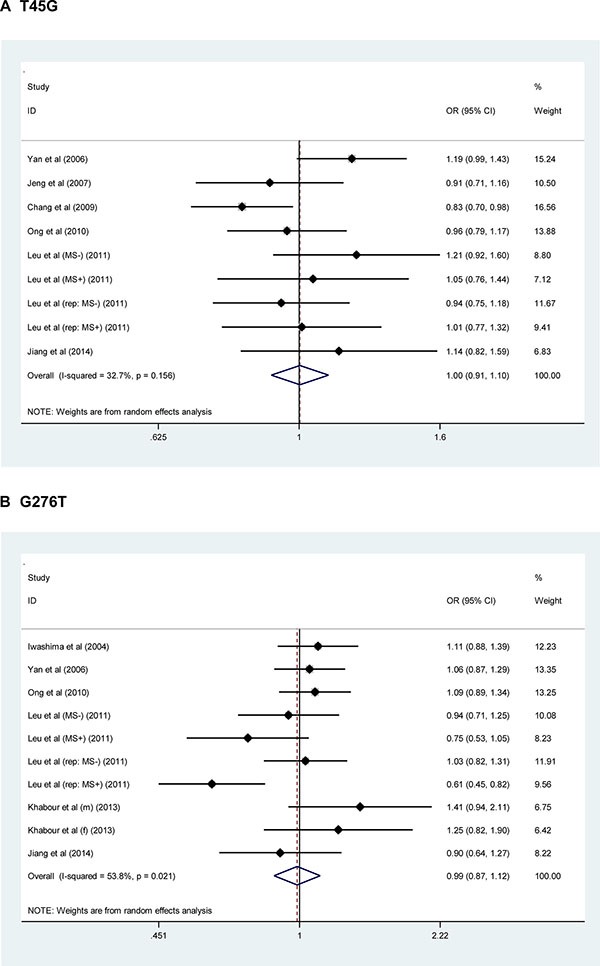
Forest plots of *ADIPOQ* two study polymorphisms in association with hypertension risk under the allelic model The right side of x-coordinate represents the increased hypertension risk, and the left side of x-coordinate represents the reduced hypertension risk. *Abbreviations*: OR, odds ratio; 95% CI, 95% confidence interval.

**Figure 2:**
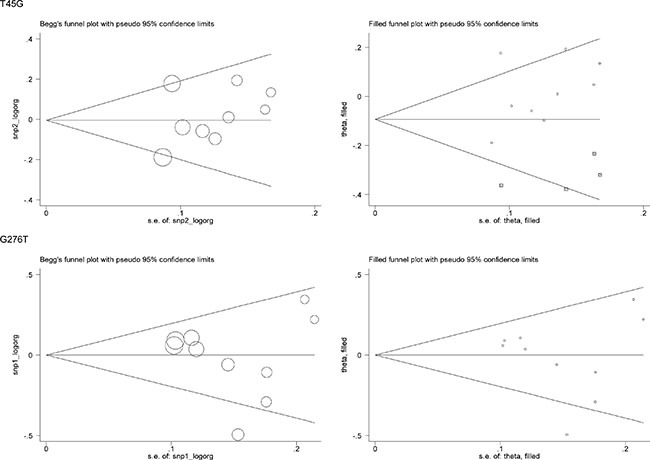
Begg's funnel plots and filled funnel plots of *ADIPOQ* two study polymorphisms in association with hypertension risk under the allelic model

For a comprehensive evaluation, the risk prediction of two study polymorphisms in *ADIPOQ* for hypertension was calculated under allelic (mutant allele versus wild allele, Figure 1), heterozygous genotypic (heterozygote versus wild homozygote, Supplementary Figure 1), homozygous genotypic (mutant homozygote versus wild homozygote, Supplementary Figure 2) and dominant (heterozygote plus mutant homozygote versus wild homozygote, Supplementary Figure 3) models, respectively. On the basis of all qualified studies, there was no indication of association between T45G or G276T polymorphism and hypertension risk under all genetic models mentioned above. Between-study heterogeneity was not significant for T45G polymorphism (*I*^2^ = 32.7%), but marginally significant for G276T polymorphism (*I*^2^ = 53.8%). The probability of publication bias was low under all genetic models for both polymorphisms, as shown by the Egger's test (all *P* > 0.10). However, there were estimated 4 missing studies to make the filled funnel plot of T45G polymorphism symmetrical, and no missing studies were reported for G276T polymorphism (Figure 2).

**Figure 3:**
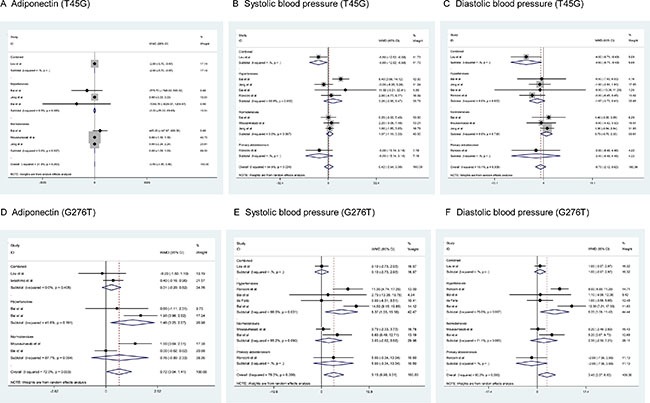
Forest plots of *ADIPOQ* two study polymorphisms for the changes of circulating adiponectin, systolic and diastolic blood pressure under the heterozygote genotypic model The right side of x-coordinate represents the increased level of phenotypes under study, and the left side of x-coordinate represents the reduced level of phenotypes under study. *Abbreviations*: WMD, weighted mean difference; 95% CI, 95% confidence interval.

**Figure 4:**
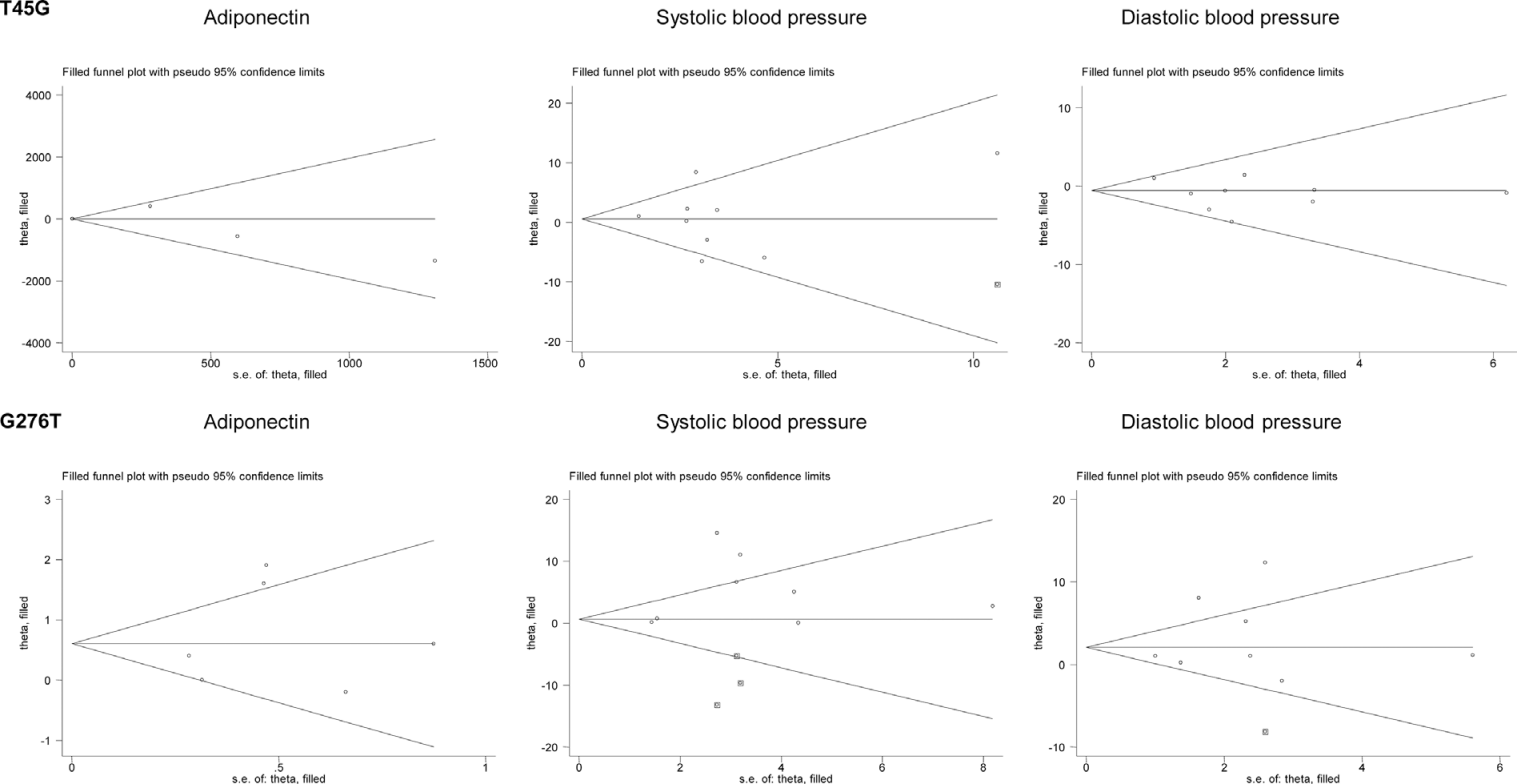
Filled funnel plots of *ADIPOQ* two study polymorphisms for the changes of circulating adiponectin, systolic and diastolic blood pressure under the heterozygote genotypic model

For the changes of adiponectin and blood pressure, only heterozygous genotypic model was summarized as the majority of enrolled studies provided their mean values only in carriers of wild homozygotes and heterozygotes (Figure 3). Overall changes were nonsignificant for T45G polymorphism. In contrast, carriers of 276GT genotype had significantly higher levels of adiponectin (weighted mean difference or WMD = 0.72 μg/mL, 95% confidence interval or CI: 0.04 to 1.41, *P* = 0.038), systolic (WMD = 5.15 mm Hg, 95% CI: 0.98 to 9.32, *P* = 0.016) and diastolic (WMD = 3.45 mm Hg, 95% CI: 0.37 to 6.53, *P* = 0.028) blood pressure than those with 276GG genotype, with evident heterogeneity (*I*^2^ = 72.0%, 78.3% and 80.0%, respectively) (Figure 3). There was no observable publication bias for both polymorphisms (Egger's test P_T45G_ = 0.574, 0.915 and 0.255 for adiponectin, systolic and diastolic blood pressure; P_G276T_ = 0.578, 0.235 and 0.505, respectively). As presented in Figure 4, there were three missing studies for the changes of systolic blood pressure across G276T genotypes, and there was one missing study for the changes of systolic blood pressure across T45G genotype and diastolic blood pressure across G276T genotypes.

### Subgroup analyses

For genetic hypertension susceptibility, a set of subgroup analyses were conducted respectively under allelic (Table 1), heterozygous genotypic (Supplementary Table 2), homozygous genotypic (Supplementary Table 3) and dominant (Supplementary Table 4) models in order to look for certain potential sources of clinical heterogeneity based on race, complicated condition, matched status, repeated measure of blood pressure, source of normotensive controls, genotyping method and total sample size (at the median cutoff of 600), respectively. For T45G polymorphism, genotyping method might be a significant source of heterogeneity, as after restricting analysis to the studies with RFLP technique, the 45G allele was associated with a 15% reduced risk of hypertension relative to the 45T allele (odds ratio or OR = 0.85, 95% CI: 0.67–0.99, *P* = 0.037). Significance was also noted in studies with RFLP technique for the comparison of the 45TG genotype with the 45TT genotype (OR = 0.81, 95% CI: 0.67–0.99, *P* = 0.037), and the 45TG+45GG genotypes with the 45TT genotype (OR = 0.80, 95% CI: 0.66–0.97, *P* = 0.020), with null heterogeneity (*I*^2^ = 0.0% for all comparisons). No significance was detected in the other subgroups. For G276T polymorphism, besides genotyping method, metabolic syndrome complication and repeated blood pressure measurement were two significant sources of between-study heterogeneity. For instance, in subjects complicated with metabolic syndrome, the 276T allele and the 276TT genotype were associated with a 33% (OR = 0.67, 95% CI: 0.53–0.83, *P* < 0.001) and 58% (OR = 0.42, 95% CI: 0.22–0.79, *P* = 0.008) reduced risk of hypertension relatively to the 276G allele and 276GG genotype, respectively, with null heterogeneity (*I*^2^ = 0.0% for both comparisons).

**Table 1:** Subgroup analyses of ADIPOQ two study polymorphisms in association with hypertension risk under the allelic model

Subgroups	T45G polymorphism			G276T polymorphism		
	s.s.	OR, 95% CI,*P*	*I*^2^ (P)	s.s.	OR, 95% CI,*P*	*I*^2^ (P)
Race						
Chinese	9	1.00, 0.91–1.10, 0.960	32.7% (0.156)	7	0.92, 0.79–1.07, 0.271	56.5% (0.032)
Jordanian	0	NA	NA	2	1.33, 0.99–1.78, 0.057	0.0% (0.679)
Complicated condition						
Metabolic syndrome	2	1.03, 0.84–1.26, 0.816	0.0% (0.858)	2	0.67, 0.53–0.83, < 0.001	0.0% (0.384)
NO	5	1.00, 0.90–1.12, 0.987	0.0% (0.481)	5	1.04, 0.87–1.29, 0.502	0.0% (0.786)
Type 2 diabetes mellitus	0	NA	NA	2	1.33, 0.99–1.78, 0.057	0.0% (0.679)
Matched status						
YES	3	1.05, 0.89–1.24, 0.581	38.4% (0.197)	2	0.81, 0.47–1.40, 0.453	88.9% (0.003)
NO	6	0.98, 0.87–1.10, 0.682	29.0% (0.218)	8	1.04, 0.93–1.16, 0.504	14.0% (0.321)
Repeated measure of BP						
NA	5	1.01, 0.91–1.13, 0.848	0.0% (0.683)	5	0.88, 0.72–1.09, 0.244	67.6% (0.015)
YES	4	1.00, 0.82–1.21, 0.961	68.2% (0.024)	5	1.10, 0.97–1.24, 0.151	0.0% (0.517)
Source of controls						
Hospital	6	1.03, 0.89–1.20, 0.698	56.2% (0.044)	6	1.01, 0.86–1.18, 0.908	32.5% (0.192)
Population	3	0.97, 0.85–1.10, 0.600	0.0% (0.927)	4	0.95, 0.75–1.21, 0.686	75.0% (0.007)
Genotyping method						
MassARRAY	2	1.01, 0.85–1.19, 0.944	0.0% (0.375)	2	1.04, 0.87–1.24, 0.675	0.0% (0.337)
RFLP	2	0.85, 0.74–0.98, 0.025	0.0% (0.546)	2	1.33, 0.99–1.79, 0.057	0.0% (0.679)
TaqMan	4	1.04, 0.91–1.18, 0.618	0.0% (0.590)	5	0.88, 0.71–1.09, 0.257	67.3% (0.016)
Total sample size*						
< 600	4	1.00, 0.87–1.16, 0.955	0.0% (0.724)	5	0.92, 0.68–1.24, 0.577	72.3% (0.006)
≥ 600	5	1.01, 0.86–1.17, 0.951	62.0% (0.032)	5	1.06, 0.96–1.17, 0.283	0.0% (0.916)

*Abbreviations*: s.s., sample size; BP, blood pressure; OR, odds ratio; 95% CI, 95% confidence interval; RFLP, restriction fragment length polymorphism; NA, not available. *The cutoff point for total sample size is based on its median value.

For the phenotypic changes across genotypes, subgroup analyses were only conducted by the status of subjects under the heterozygous genotypic model (Figure 3). No significance was observed for T45G polymorphism across subgroups. In contrast, the mean levels of circulating adiponection (WMD = 1.46 μg/mL, 95% CI: 0.25 to 2.67, *P* = 0.018), systolic (WMD = 8.37 mm Hg, 95% CI: 1.55 to 15.18, *P* = 0.016) and diastolic (WMD = 6.25 mm Hg, 95% CI: 1.08 to 11.42, *P* = 0.018) blood pressure were increased significantly in hypertensive patients, but with moderate or strong heterogeneity (*I*^2^ = 41.6%, 66.3% and 75.0%, respectively).

### Meta-regression analyses

In order to look for additional sources of heterogeneity, meta-regression analyses were conducted by incorporating age, sex, body mass index, total cholesterol, triglycerides, high-density lipoprotein cholesterol, low-density lipoprotein cholesterol and fasting blood glucose as covariates, while none of them exhibited an obvious confounding influence on the association between the two examined polymorphisms and hypertension risk (*P* > 0.05 for testing the significance of regression coefficients).

## DISCUSSION

This meta-analysis was prepared to synthesize available published data on the association of two widely evaluated polymorphisms in *ADIPOQ* with hypertension risk and the changes of circulating adiponectin and blood pressure. The key findings of this meta-analysis suggested that in spite of the nonsignificant association between *ADIPOQ* T45G or G276T polymorphism and hypertension, the heterozygous mutation of G276T was observed to account for increased levels of circulating adiponectin and blood pressure, especially in hypertensive patients. Our findings collectively support the proposition that the elevated hypertension risk driven by a single locus is small, although the modulatory impact of this locus on certain intermediate phenotypes might be significant.

The negative findings of T45G and G276T polymorphisms in predisposition to hypertension were in agreement with the findings of a previous meta-analysis by Xi et al. who synthesized only Chinese subjects [23]. Although the majority of studies in the current meta-analysis were from China, our subgroup analyses dropped a hint that race might explain genetic heterogeneity as the relationship between G276T and hypertension risk represented an opposite tendency between Chinese and other racial groups (Japanese and Jordanians), albeit no statistical significance was attained. It could be speculated that the linkage disequilibrium structure of human *ADIPOQ* may vary in distinct racial groups. The lack of a consistent association across different races between G276T polymorphism at intron 2 and hypertension risk has suggested that another locus in linkage disequilibrium with this polymorphism may be the causal mutation. Further unearthing of the genetic structure of *ADIPOQ* might enhance our understanding on the genetic basis of hypertension. In addition, there is compelling evidence that hypertension is a complex disorder, to which genetic and environmental factors contribute interactively [24]. For instance, after treating male Sprague-Dawley rats with high-salt diets for 5 weeks, salt loading was observed to independently increase circulating adiponectin concentration, suggesting a regulatory role of salt intake in adiponectin [25]. A family-based association study in Chinese has shown that genetic defects in *ADIPOQ* may attribute to the development of salt sensitivity and potassium sensitivity of blood pressure [26]. Besides genetic heterogeneity, a growing focus on the sub-phenotypes of hypertension such as salt-sensitive hypertension and low-renin hypertension has become increasingly attractive. Noteworthily, overlooking potential gene-environment interaction will likely mask the detection of genetic culprits with small effect and may lead to inconclusive findings across studies, just as the current meta-analysis does.

However, there is a note of caution to interpret the changes of circulating adiponectin and blood pressure across G276T genotypes in the current meta-analysis. It seems somewhat counterintuitive for the simultaneous increases in adiponectin and blood pressure among carriers of 276GT genotype relative to the 276GG homozygotes. Adiponectin has insulin-sensitizing anti-inflammation and anti-atherogenic properties, and it hence exerts a protective role in type 2 diabetes mellitus, obesity, hypertension and coronary artery disease [27, 28]. It is kind of contradiction for the parallel changes between adiponectin and blood pressure across G276T genotypes. One possible explanation is the confounding impact of antihypertensive agents, as renin-angiotensin system inhibitors have been proven to increase adiponectin secretion, while reducing blood pressure [29]. Based on the definition of hypertension among individual studies of this meta-analysis, patients receiving antihypertensive therapy were classified as having hypertension in almost all studies. However, information on the type and dose of antihypertensive agents and the percentage of treated patients was rarely reported in these studies. It is therefore tempting to examine the changes of adiponectin and blood pressure among untreated hypertensive patients. Also, as a support of this explanation, the increases of adiponectin and blood pressure were reinforced after restricting analysis to hypertensive patients. Nevertheless in the current meta-analysis, the clinical implication of our findings remains speculative. However, if our findings can be validated by other larger, well-designed, prospective studies in the future investigations, then it is expected that the impact of *ADIPOQ* G276T polymorphism, if involved, could perhaps guide the development of effective therapeutic interventions.

Several limitations should be acknowledged for the current meta-analysis. The first was the literature coverage, as published articles in languages other than English were not considered for analysis, which is susceptible to a local literature bias. The second limitation was that as with a majority of meta-analyses, heterogeneity is a chief issue, especially when the number of studies is limited and/or their individual statistical power is low. Although we tried very hard to detect certain potential sources of heterogeneity through subgroup and meta-regression analyses, some comparisons were stilled nagged by evident heterogeneity. The third limitation was that it was premature to translate our findings into a clinical recommendation given the insufficient study power of this study. The fourth limitation was that all studies in the current meta-analysis were from Asian countries, and it was of added interest to confirm our findings in other continental groups.

Taken together, our meta-analytical findings suggested that in spite of the nonsignificant association between *ADIPOQ* T45G or G276T polymorphism and hypertension, the heterozygous mutation of G276T was observed to account for increased levels of circulating adiponectin and blood pressure, especially in hypertensive patients. However, whether this polymorphism may prove to be of clinical value in hypertensive patients remains to be determined. Future studies seeking to illustrate the biological or clinical potentials of *ADIPOQ* genetic defects in the pathogenesis of hypertension are very much needed.

## MATERIALS AND METHODS

### PRISMA guideline

The conduct of this meta-analysis accords with the guidelines from the PRISMA (the preferred reporting items for systematic reviews and meta-analyses) statement [30], as presented in the Supplementary Table 5.

### Literature search

Two public databases, MEDLINE and EMBASE, were searched from the date of creation to October 8, 2016 for all potential articles that were published in English language. The predefined subject headings included (“adiponectin” OR “ADIPOQ” OR “APM-1” OR “APM1”) AND (“hypertension” OR “blood pressure”) AND (“polymorphism” OR “variant” OR “allele” OR “genotype”). Beyond this, the reference lists of some original articles or reviewers were also inspected for likely missing hits during the subject-heading search. The literature search was done in duplicate by two investigators (Jianmin Wu and Guoyan Xu), and the finally retrieved articles were managed by the EndNote X5 software (available at the website: http://www.endnote.com).

### Inclusion criteria

An article was qualified for analysis if the following criteria were satisfied: (i) T45G and/or G276T polymorphisms were genotyped by a valid method; (ii) for hypertension risk, the genotype or allele counts of study polymorphism(s) were provided between hypertensive and normotensive subjects; (iii) for the genotype-phenotype relationship, the mean value and standard deviation or derivates (such as standard error, 95% confidence interval or range) of circulating adiponectin or systolic/diastolic blood pressure were provided.

### Exclusion criteria

An article was excluded if it was a conference abstract or poster, case report, editorial comment, narrative or systematic review. In addition, only primary hypertension was considered, and other types of hypertension such as gestational hypertension were excluded. If data or sub-data of the same study group were published more than once, only the complete or latest article was retained for meta-analysis.

### Data collection

With abovementioned inclusion/exclusion criteria in mind, the two investigators (Jianmin Wu and Guoyan Xu) independently collected data from each qualified article. The data were typed into a pre-determined Excel format that covered information on the first author's surname, publication year, country, race, sample size, associated complication, matched status, repeated measure of blood pressure (yes or no), source of normotensive controls (population-based or hospital-based), genotyping method, diagnosis of hypertension, as well as anthropometric indices (age, sex, body mass index, systolic and diastolic blood pressure), clinical markers (fasting blood glucose, triglycerides, total cholesterol, high-density lipoprotein cholesterol, low-density lipoprotein cholesterol and adiponectin), genetic distributions (genotype or allele counts of T45G and/or G276T polymorphisms between hypertensive and normotensive subjects) and phenotype changes (mean and standard deviation of circulating adiponectin and/or blood pressure across genotypes). Here, population-based controls referred to the normotensive subjects enrolled from either general populations, communities, volunteers or medical examination centers. The two independent Excel forms were compared for divergence by the self-compiled computer program, and a universe consensus was reached finally between the two investigators who were in charge of data collection.

### Statistical analyses

The risk prediction of study polymorphisms for hypertension was measured by OR with 95% CI. The changes of circulating adiponectin and blood pressure between genotypes were measured by WMD with 95% CI. Given the fact that risk estimates are almost equal in case of no heterogeneity between fixed-effects and random-effects models, individual ORs or WMDs were pooled unanimously under the random-effects model with the DerSimonian-Laird method. The magnitude of between-study heterogeneity was marked by the *I*^2^ statistic, which ranges from 0% to 100% with a higher value denoting a higher likelihood of heterogeneity. In fact, the *I*^2^ statistic is a transformation of Q statistic, as 100% × (Q- degree of freedom)/Q and it estimates the percentage of the variation in effect sizes that is due to heterogeneity [31]. Usually, a cutoff point of 50% was generally used to delimit the significance of between-study heterogeneity [31].

The contribution of individual studies to pooled estimate was assessed by a sensitivity analysis, which omitted each study one by one and calculated the differential estimate of the other studies. Potential sources of heterogeneity were determined by both subgroup analyses and meta-regression analyses.

The possibility that some small studies with negative findings are not easily to be published is termed as publication bias, which was justified by the Egger's test and the filled funnel plots. For the Egger's test, the probability of 10% was chosen as a significant cutoff point. The filled funnel plots were generated by the trim-and-fill method, which can estimate the number of putative missing studies.

Data analyses were done by the Stata software version 12.0 for Windows (StataCorp, College Station, Texas, USA).

## SUPPLEMENTARY MATERIALS FIGURES AND TABLES






