# Supplementary material for: The association of two polymorphisms in adiponectin-encoding gene with hypertension risk and the changes of circulating adiponectin and blood pressure: A meta-analysis
Source: Oncotarget. 2017 Jan 16;8(9):14636–45. doi: 10.18632/oncotarget.14680 (PMC5362431; doi:10.18632/oncotarget.14680)
Supplement: Supplementary file 1 [file oncotarget-08-14636-s001.pdf]

The association of two polymorphisms in adiponectin-encoding gene with hypertension risk and the changes of circulating adiponectin and blood pressure: A meta-analysis

Supplementary Materials

Supplementary Table 1: The baseline characteristics of all eligible studies in association with hypertension risk. See Supplementary_Table_1

Supplementary Table 2: Subgroup analyses of ADIPOQ two study polymorphisms in association with hypertension risk under the heterozygote genotypic model

Subgroups	T45G polymorphism			G276T polymorphism		
Race	s.s.	OR, 95% CI, P	I^2 (P)	s.s.	OR, 95% CI, P	I^2 (P)
Chinese	9	0.93, 0.84–1.04, 0.211	0.0% (0.592)	7	0.89, 0.72–1.09, 0.250	60.9% (0.018)
Jordanian	0	NA	NA	2	1.35, 0.92–1.99, 0.130	0.0% (0.553)
Complicated condition						
Metabolic syndrome	2	0.97, 0.83–1.13, 0.850	0.0% (0.721)	2	0.72, 0.41–1.25, 0.239	72.8% (0.055)
NO	5	0.97, 0.83–1.13, 0.686	0.0% (0.463)	5	0.97, 0.80–1.18, 0.766	40.6% (0.151)
Type 2 diabetes	0	NA	NA	2	1.35, 0.92–1.99, 0.130	0.0% (0.553)
Matched status						
YES	3	0.98, 0.81–1.18, 0.808	0.0% (0.813)	2	0.77, 0.41–1.44, 0.404	86.9% (0.006)
NO	6	0.92, 0.80–1.06, 0.259	13.3% (0.329)	8	1.02, 0.88–1.19, 0.793	24.4% (0.234)
Repeated measure of BP						
NA	5	0.96, 0.83–1.12, 0.619	0.0% (0.619)	5	0.91, 0.71–1.17, 0.454	62.1% (0.032)
YES	4	0.91, 0.77–1.08, 0.292	15.5% (0.314)	5	1.02, 0.79–1.31, 0.904	51.9% (0.081)
Source of controls						
Hospital	6	0.95, 0.82–1.09, 0.457	10.1% (0.351)	6	0.98, 0.78–1.22, 0.821	40.5% (0.135)
Population	3	0.92, 0.77–1.10, 0.371	0.0% (0.636)	4	0.93, 0.69–1.25, 0.645	72.8% (0.012)
Genotyping method						
MassARRAY	2	1.02, 0.81–1.28, 0.896	0.0% (0.416)	2	0.82, 0.44–1.51, 0.521	81.6% (0.020)
RFLP	2	0.81, 0.67–0.99, 0.037	0.0% (0.614)	2	1.35, 0.92–1.99, 0.130	0.0% (0.553)
TaqMan	4	0.97, 0.81–1.16, 0.701	0.0% (0.450)	5	0.90, 0.70–1.16, 0.416	59.9% (0.041)
Total sample size						
< 600	4	0.99, 0.81–1.20, 0.885	0.0% (0.758)	5	0.86, 0.59–1.28, 0.462	71.4% (0.007)
≥ 600	5	0.92, 0.80–1.06, 0.245	18.8% (0.295)	5	1.05, 0.92–1.20, 0.451	0.0% (0.939)

Abbreviations: s.s., sample size; BP, blood pressure; OR, odds ratio; 95% CI, 95% confidence interval; RFLP, restriction fragment length polymorphism; NA, not available.

Supplementary Table 3: Subgroup analyses of ADIPOQ two study polymorphisms in association with hypertension risk under the homozygote genotypic model

Subgroups	T45G polymorphism			G276T polymorphism		
Race	s.s.	OR, 95% CI, P	I^2 (P)	s.s.	OR, 95% CI, P	I^2 (P)
Chinese	9	1.06, 0.88–1.28, 0.552	10.4% (0.348)	7	0.93, 0.67–1.29, 0.657	37.3% (0.141)
Jordanian	0	NA	NA	2	1.96, 0.88–4.37, 0.099	0.0% (0.937)
Complicated condition						
Metabolic syndrome	2	1.13, 0.69–1.87, 0.622	0.0% (0.583)	2	0.42, 0.22–0.79, 0.008	0.0% (0.593)
NO	5	1.03, 0.80–1.34, 0.809	0.0% (0.702)	5	1.16, 0.88–1.52, 0.301	0.0% (0.763)
Type 2 diabetes	0	NA	NA	2	1.96, 0.88–4.37, 0.099	0.0% (0.937)
Matched status						
YES	3	1.13, 0.79–1.62, 0.515	38.2% (0.198)	2	0.82, 0.37–1.84, 0.631	62.2% (0.104)
NO	6	0.98, 0.78–1.23, 0.868	0.0% (0.459)	8	1.11, 0.82–1.52, 0.500	29.1% (0.196)
Repeated measure of BP						
NA	5	1.10, 0.84–1.44, 0.480	0.0% (0.815)	5	0.79, 0.53–1.19, 0.266	42.3% (0.140)
YES	4	1.01, 0.69–1.48, 0.945	58.4% (0.065)	5	1.36, 1.00–1.86, 0.053	0.0% (0.810)
Source of controls						
Hospital	6	1.10, 0.82–1.47, 0.534	41.4% (0.129)	6	1.11, 0.71–1.74, 0.659	45.2% (0.104)
Population	3	1.00, 0.73–1.37, 0.995	0.0% (0.899)	4	1.03, 0.73–1.45, 0.880	20.9% (0.285)
Genotyping method						
MassARRAY	2	0.99, 0.66–1.51, 0.976	0.0% (0.551)	2	1.30, 0.84–2.02, 0.237	0.0% (0.479)
RFLP	2	0.77, 0.56–1.05, 0.102	0.0% (0.699)	2	1.96, 0.88–4.37, 0.099	0.0% (0.937)
TaqMan	4	1.19, 0.86–1.65, 0.284	0.0% (0.846)	5	0.80, 0.51–1.23, 0.305	46.5% (0.113)
Total sample size						
< 600	4	1.02, 0.73–1.42, 0.931	0.0% (0.740)	5	0.99, 0.47–2.09, 0.973	64.8% (0.023)
≥ 600	5	1.08, 0.81–1.44, 0.612	47.3% (0.108)	5	1.12, 0.87–1.44, 0.371	0.0% (0.907)

Abbreviations: s.s., sample size; BP, blood pressure; OR, odds ratio; 95% CI, 95% confidence interval; RFLP, restriction fragment length polymorphism; NA, not available.

Supplementary Table 4: Subgroup analyses of ADIPOQ two study polymorphisms in association with hypertension risk under the dominant model

Subgroups	T45G polymorphism			G276T polymorphism		
Race	s.s.	OR, 95% CI, P	I^2 (P)	s.s.	OR, 95% CI, P	I^2 (P)
Chinese	9	0.96, 0.86–1.08, 0.500	13.8% (0.320)	7	0.89, 0.73–1.08, 0.234	59.4% (0.022)
Jordanian	0	NA	NA	2	1.41, 0.97–2.06, 0.071	0.0% (0.576)
Complicated condition						
Metabolic syndrome	2	1.00, 0.85–1.13, 0.987	0.0% (0.885)	2	0.66, 0.44–1.00, 0.049	55.8% (0.133)
NO	5	0.98, 0.85–1.13, 0.797	0.0% (0.414)	5	1.02, 0.88–1.17, 0.842	8.1% (0.360)
Type 2 diabetes	0	NA	NA	2	1.41, 0.97–2.06, 0.071	0.0% (0.576)
Matched status						
YES	3	1.03, 0.86–1.23, 0.765	0.0% (0.494)	2	0.77, 0.40–1.48, 0.427	88.9% (0.003)
NO	6	0.94, 0.81–1.10, 0.446	28.2% (0.223)	8	1.03, 0.90–1.19, 0.643	17.0% (0.296)
Repeated measure of BP						
NA	5	0.98, 0.85–1.13, 0.816	0.0% (0.614)	5	0.88, 0.68–1.14, 0.335	66.4% (0.018)
YES	4	0.96, 0.77–1.19, 0.677	52.8% (0.096)	5	1.07, 0.86–1.33, 0.535	37.8% (0.169)
Source of controls						
Hospital	6	0.99, 0.84–1.18, 0.946	41.8% (0.127)	6	0.99, 0.81–1.21, 0.918	34.4% (0.179)
Population	3	0.93, 0.79–1.11, 0.427	0.0% (0.757)	4	0.94, 0.69–1.27, 0.665	76.1% (0.006)
Genotyping method						
MassARRAY	2	1.01, 0.82–1.26, 0.910	0.0% (0.367)	2	0.90, 0.57–1.43, 0.660	70.5% (0.066)
RFLP	2	0.80, 0.66–0.97, 0.020	0.0% (0.594)	2	1.41, 0.97–2.06, 0.071	0.0% (0.576)
TaqMan	4	1.00, 0.84–1.19, 0.980	0.0% (0.461)	5	0.88, 0.68–1.14, 0.323	64.8% (0.023)
Total sample size						
< 600	4	1.00, 0.82–1.20, 0.957	0.0% (0.725)	5	0.88, 0.60–1.28, 0.500	71.8% (0.007)
≥ 600	5	0.96, 0.81–1.14, 0.625	48.3% (0.102)	5	1.06, 0.94–1.21, 0.343	0.0% (0.929)

Abbreviations: s.s., sample size; BP, blood pressure; OR, odds ratio; 95% CI, 95% confidence interval; RFLP, restriction fragment length polymorphism; NA, not available.

Supplementary Table 5: The PRISMA checklist. See Supplementary_Table_5

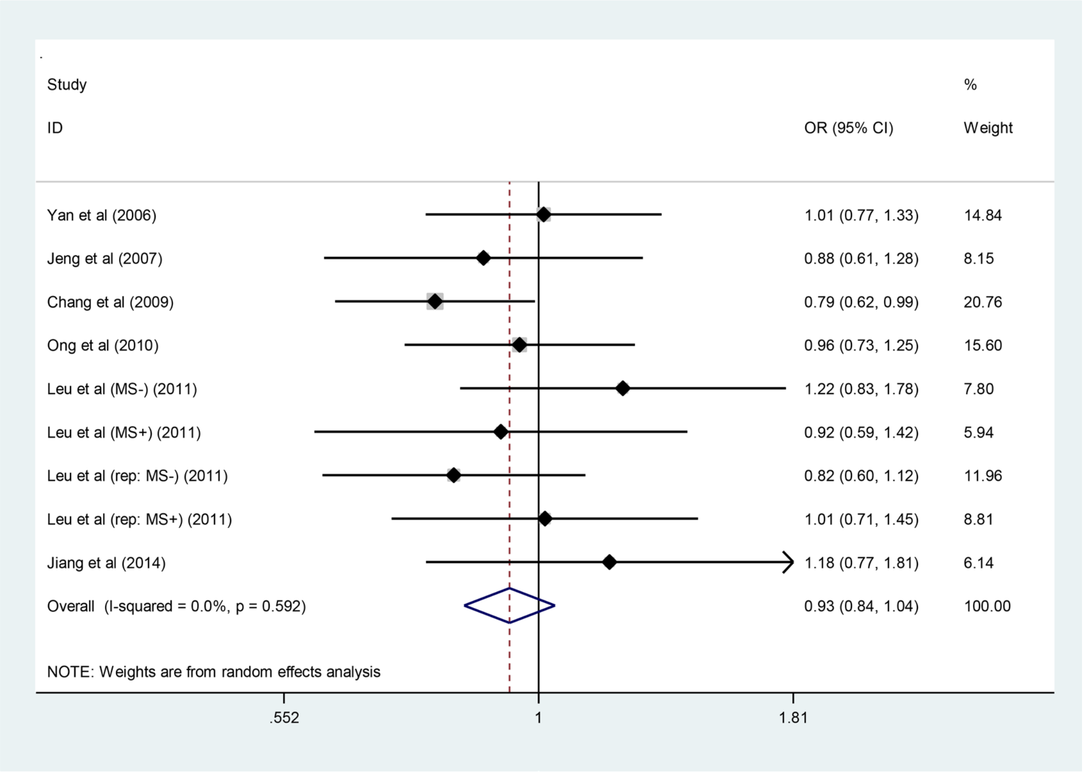

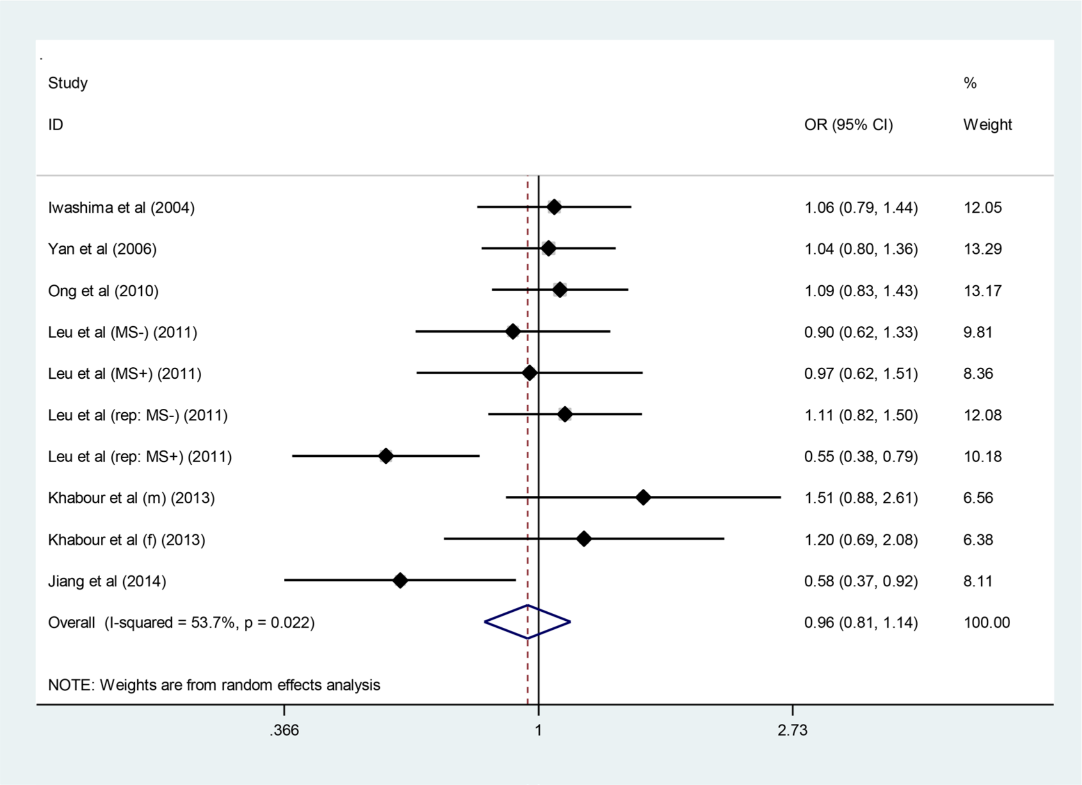

Supplementary Figure 1: Forest plots of ADIPOQ two study polymorphisms in association with hypertension risk under the heterozygous genotypic model.

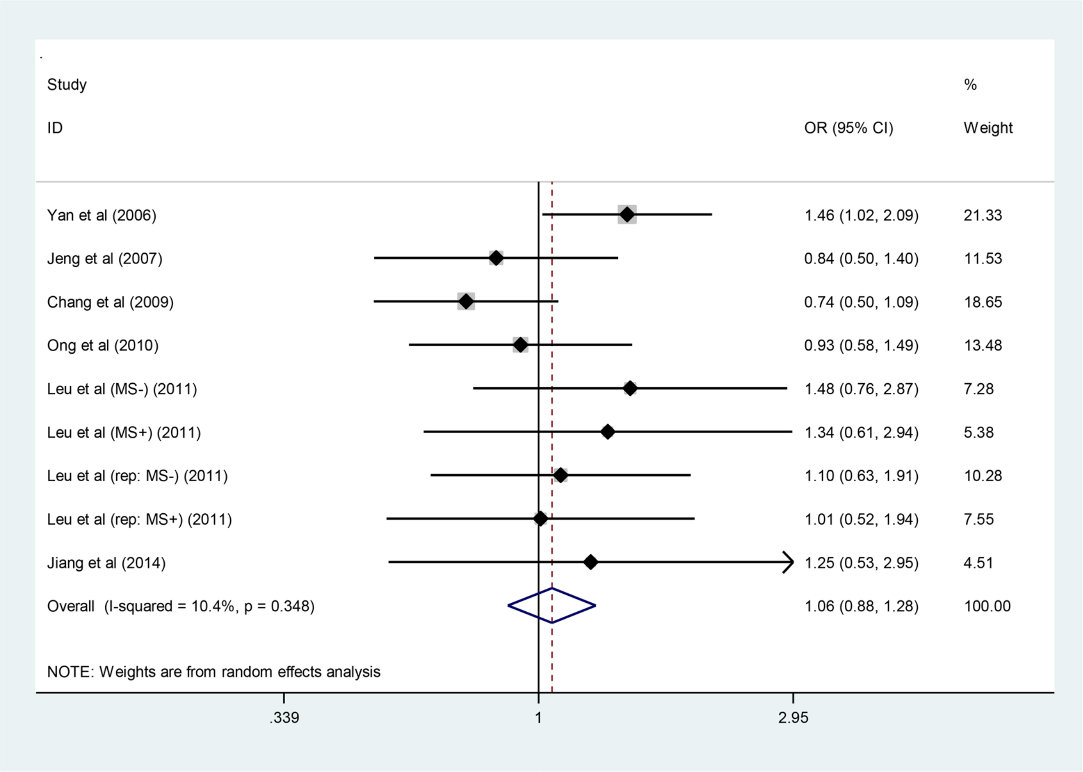

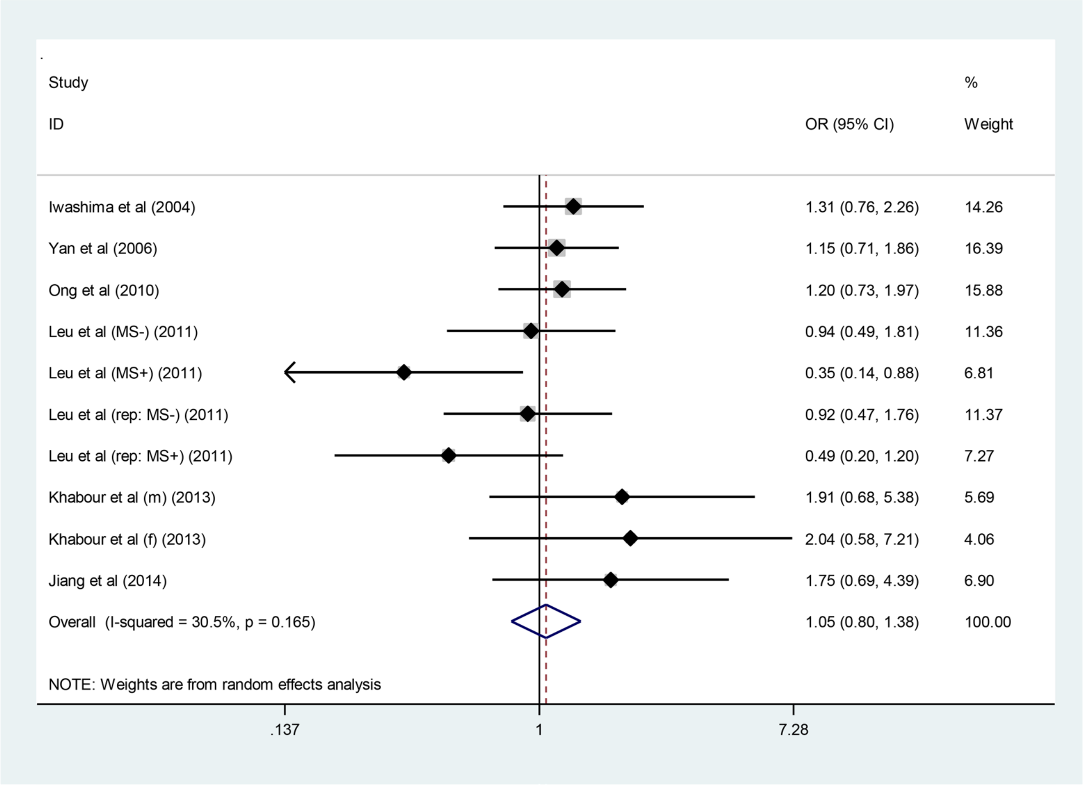

Supplementary Figure 2: Forest plots of ADIPOQ two study polymorphisms in association with hypertension risk under the homozygous genotypic model.

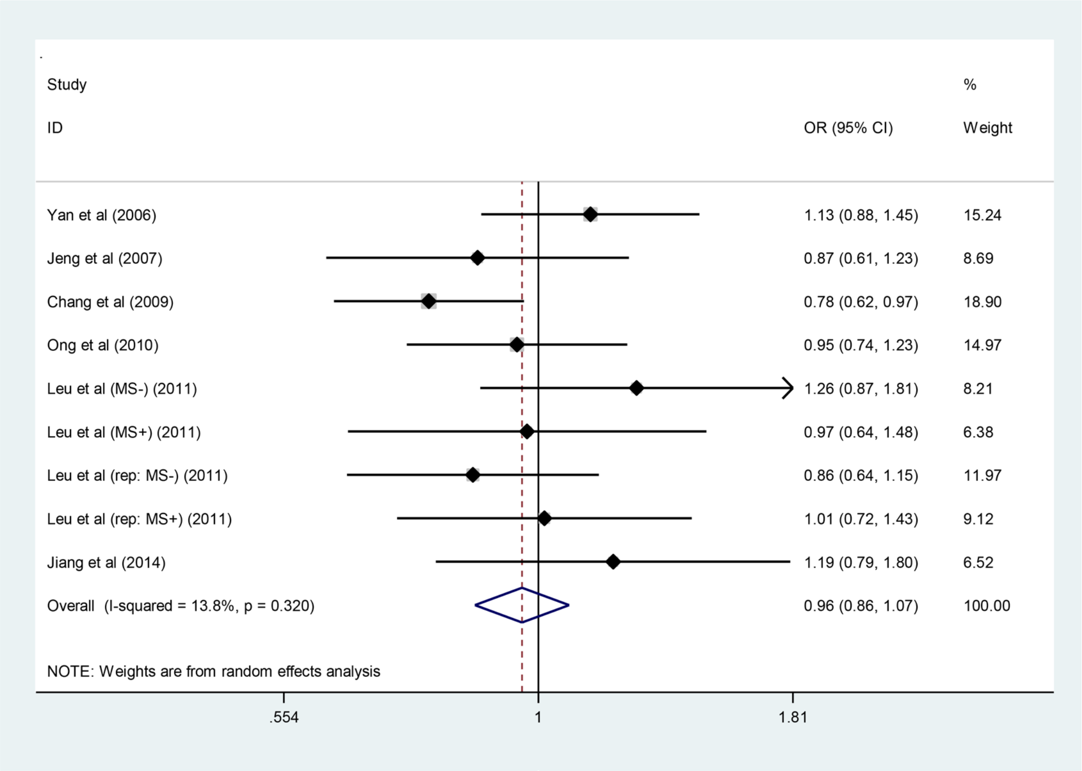

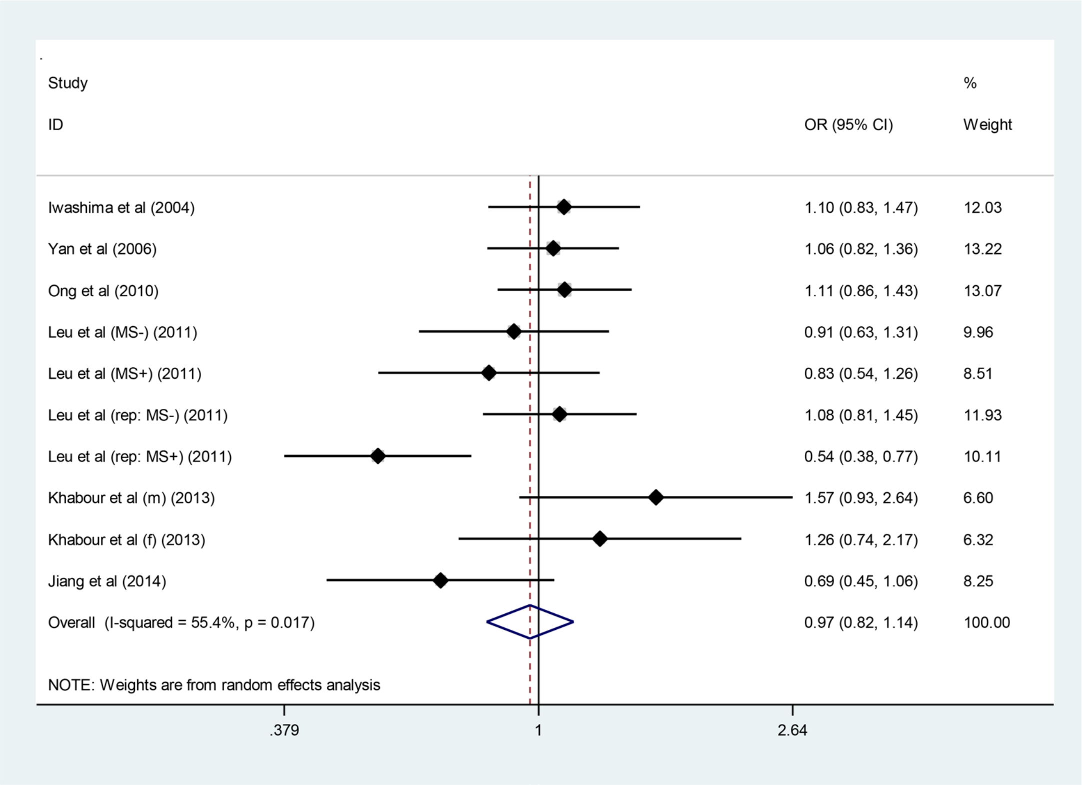

Supplementary Figure 3: Forest plots of ADIPOQ two study polymorphisms in association with hypertension risk under the dominant model.
