# Supplementary material for: The association of two polymorphisms in adiponectin-encoding gene with hypertension risk and the changes of circulating adiponectin and blood pressure: A meta-analysis
Source: Oncotarget. 2017 Jan 16;8(9):14636–45. doi: 10.18632/oncotarget.14680 (PMC5362431; doi:10.18632/oncotarget.14680)
Supplement: Supplementary file 2 [file oncotarget-08-14636-s002.docx]

**Supplementary Table 1.** The baseline characteristics of all eligible studies in association with hypertension risk

| **First author (year)** | **Race** | **Complicated** | **Matched** | **Repeated BP measure** | **Source of controls** | **Genotyping** | **Diagnosis** |
| --- | --- | --- | --- | --- | --- | --- | --- |
| Iwashima et al (2004) | Japanese | NO | No | YES | Population | TaqMan | SBP/DBP>=140/90 mm Hg or on antihypertensive treatment |
| Yan et al (2006) | Chinese | Obesity | Yes | YES | Hospital | Sequencing | SBP/DBP>=160/90 mm Hg or on antihypertensive treatment |
| Jeng et al (2007) | Chinese | NO | Yes | YES | Hospital | RFLP | SBP/DBP>160/95 mm Hg or on antihypertensive treatment |
| Chang et al (2009) | Chinese | CAD | No | YES | Hospital | RFLP | SBP/DBP>=140/90 mm Hg |
| Ong et al (2010) | Chinese | NO | No | NA | Population | MassARRAY | NA |
| Leu et al (MS-) (2011) | Chinese | NO | No | NA | Hospital | TaqMan | SBP/DBP>140/90 mm Hg or on antihypertensive treatment |
| Leu et al (MS+) (2011) | Chinese | Metabolic syndrome | No | NA | Hospital | TaqMan | SBP/DBP>140/90 mm Hg or on antihypertensive treatment |
| Leu et al (rep: MS-) (2011) | Chinese | NO | No | NA | Population | TaqMan | SBP/DBP>140/90 mm Hg or on antihypertensive treatment |
| Leu et al (rep: MS+) (2011) | Chinese | Metabolic syndrome | Yes | NA | Population | TaqMan | SBP/DBP>140/90 mm Hg or on antihypertensive treatment |
| Khabour et al (m) (2013) | Jordanian | Type 2 diabetes | No | YES | Hospital | RFLP | SBP/DBP>140/90 mm Hg or on antihypertensive treatment |
| Khabour et al (f) (2013) | Jordanian | Type 2 diabetes | No | YES | Hospital | RFLP | SBP/DBP>140/90 mm Hg or on antihypertensive treatment |
| Jiang et al (2014) | Chinese | NO | No | YES | Hospital | MassARRAY | SBP/DBP>140/90 mm Hg or on antihypertensive treatment |

**(Cont’d)**

| **First author (year)** | **Sample size** | | **Age (years)** | | **Males** | | **BMI (kg/m^2^)** | | **SBP (mm Hg)** | | **DBP (mm Hg)** | |
| --- | --- | --- | --- | --- | --- | --- | --- | --- | --- | --- | --- | --- |
|  | **Cases** | **Controls** | **Cases** | **Controls** | **Cases** | **Controls** | **Cases** | **Controls** | **Cases** | **Controls** | **Cases** | **Controls** |
| Iwashima et al (2004) | 446 | 312 | 59.4 | 57.1 | 1.0000 | 1.0000 | 24.4 | 23.1 | 138 | 119 | 83 | 72 |
| Yan et al (2006) | 494 | 502 | 49 | 48 | 0.5526 | 0.4163 | 30.8 | 22.4 | 144.5 | 116.7 | 94.9 | 74.6 |
| Jeng et al (2007) | 212 | 356 | 51.6 | 50.7 | 0.5802 | 0.5197 | 25.7 | 23.8 | 151 | 117 | 94 | 76 |
| Chang et al (2009) | 560 | 727 | NA | NA | 0.6472 | 0.6472 | NA | NA | NA | NA | NA | NA |
| Ong et al (2010) | 285 | 1331 | 63.2 | 48.8 | 0.4910 | 0.4400 | 26.5 | 23.5 | 153.9 | 113.9 | 86.3 | 72.1 |
| Leu et al (MS-) (2011) | 159 | 446 | 41.4 | 46.7 | 0.5660 | 0.4821 | 25.4 | 23.5 | 140.3 | 119.4 | 89 | 74.3 |
| Leu et al (MS+) (2011) | 192 | 165 | 41.7 | 57.5 | 0.6510 | 0.3939 | 28.3 | 27.1 | 140.1 | 137.7 | 90.5 | 83.3 |
| Leu et al (rep: MS-) (2011) | 246 | 667 | 57.4 | 53.3 | 0.6098 | 0.5067 | 23.5 | 23.1 | 139.2 | 111.4 | 86.4 | 71.5 |
| Leu et al (rep: MS+) (2011) | 290 | 240 | 57.8 | 56.8 | 0.4690 | 0.3417 | 26.4 | 26.4 | 141.8 | 114.8 | 86.7 | 73.2 |
| Khabour et al (m) (2013) | 112 | 101 | 56.91 | 54.22 | 1.0000 | 1.0000 | 29.47 | 31.49 | NA | NA | NA | NA |
| Khabour et al (f) (2013) | 139 | 98 | 58.14 | 53.46 | 0.0000 | 0.0000 | 33.38 | 33.1 | NA | NA | NA | NA |
| Jiang et al (2014) | 223 | 176 | 68.8 | 67.1 | 0.3004 | 0.4261 | 24.5 | 23.1 | 154.6 | 121.3 | 90.2 | 76.8 |

**(Cont’d)**

| **First author (year)** | **TC (mmol/L)** | | **TG (mmol/L)** | | **HDLC (mmol/L)** | | **LDLC (mmol/L)** | | **FBG (mmol/L)** | | **Adiponectin (μg/mL)** | |
| --- | --- | --- | --- | --- | --- | --- | --- | --- | --- | --- | --- | --- |
|  | **Cases** | **Controls** | **Cases** | **Controls** | **Cases** | **Controls** | **Cases** | **Controls** | **Cases** | **Controls** | **Cases** | **Controls** |
| Iwashima et al (2004) | 5.34 | 5.17 | 1.77 | 1.65 | 1.32 | 1.32 | NA | NA | 6.24 | 5.98 | 5.2 | 6.4 |
| Yan et al (2006) | 5.14 | 4.76 | 1.83 | 1.21 | 1.13 | 1.33 | 2.94 | 2.88 | 5.43 | 4.91 | NA | NA |
| Jeng et al (2007) | 5.20 | 5.35 | 1.80 | 1.40 | NA | NA | NA | NA | 5.72 | 5.94 | 9.7 | 11.5 |
| Chang et al (2009) | NA | NA | NA | NA | NA | NA | NA | NA | NA | NA | NA | NA |
| Ong et al (2010) | NA | NA | 1.49 | 1.09 | 1.24 | 1.37 | 3.54 | 3.24 | 5.90 | 5.14 | 6.185 | 6.77 |
| Leu et al (MS-) (2011) | 5.06 | 4.99 | 1.32 | 1.25 | 1.35 | 1.39 | 3.25 | 3.10 | 5.46 | 5.37 | 11.9 | 15.9 |
| Leu et al (MS+) (2011) | 5.57 | 5.42 | 2.72 | 2.66 | 1.06 | 1.09 | 3.30 | 3.17 | 7.08 | 7.94 | 9.3 | 13.4 |
| Leu et al (rep: MS-) (2011) | 5.06 | 5.02 | 0.94 | 0.98 | 1.26 | 1.19 | 3.50 | 3.23 | 5.17 | 5.12 | NA | NA |
| Leu et al (rep: MS+) (2011) | 5.55 | 5.58 | 1.79 | 2.16 | 0.96 | 0.92 | 3.71 | 3.75 | 5.97 | 6.21 | NA | NA |
| Khabour et al (m) (2013) | 4.77 | 4.83 | 2.28 | 2.16 | 1.01 | 1.08 | 2.94 | 2.99 | 11.25 | 9.49 | 11.09 | 12.85 |
| Khabour et al (f) (2013) | 5.28 | 5.08 | 2.64 | 2.04 | 1.26 | 1.27 | 3.00 | 3.10 | 9.39 | 8.89 | 11.62 | 12.77 |
| Jiang et al (2014) | 5.20 | 5.10 | 1.70 | 1.60 | 1.40 | 1.40 | 3.30 | 3.20 | NA | NA | NA | NA |

*Abbreviations*: BP, blood pressure; SBP, systolic blood pressure; DBP, diastolic blood pressure; NA, not available; CAD, coronary artery disease; RFLP, restriction fragment length polymorphism; BMI, body mass index; TC, total cholesterol; TG, triglycerides; HDLC, high-density lipoprotein cholesterol; LDLC, low-density lipoprotein cholesterol; FBG, fasting blood glucose.
